# Addressing health literacy among long-term unemployed persons: the FORESIGHT intervention study

**DOI:** 10.1186/s12889-025-25313-4

**Published:** 2025-11-17

**Authors:** Tilman Brand, Himal Singh, Claudia Steiner, Wiebke Hübner, Meret Lakeberg, Jonathan Kolschen, Florence Samkange-Zeeb, Benjamin Schüz, Hajo Zeeb

**Affiliations:** 1 Department of Prevention and Evaluation, Leibniz Institute for Prevention Research and Epidemiology – BIPS, Achterstr. 30, Bremen, 28359 Germany; 2 Institute for Public Health and Nursing Research, University of Bremen, Grazer Str. 4, Bremen, 28359 Germany; 3 Gröpelinger Recycling Initiative, Oslebshauser Landstraße 30, Bremen, 28239 Germany; 4 Department of Health Services Research, Carl von Ossietzky University Oldenburg, Oldenburg, 26111 Germany; 5 Health Sciences Bremen, University of Bremen, Bremen, Germany

**Keywords:** Health literacy, Food literacy, Problem-based learning, Participation, Unemployed

## Abstract

**Background:**

Long-term unemployment is associated with various health risks and low health literacy. Occupational rehabilitation organizations that employ individuals with a history of long-term unemployment may be ideal settings for promoting health literacy. The purpose of this study was to develop and test an intervention to enhance health literacy in this setting.

**Methods:**

The intervention was developed using the steps of the Intervention Mapping protocol. It focused on nutrition, physical activity, and mental health literacy, and included both problem-based learning and practical activities. The intervention was tested using a single-arm pre-post design. Health literacy was assessed at the individual level using the European Health Literacy Scale (HLS-EU-Q16) for general health literacy, the Newest Vital Sign test for functional health literacy, and domain-specific literacies for food, physical activity and mental health literacy. Additionally, structured interviews were conducted with social workers and management staff in the participating organizations to evaluate organizational readiness for change, covering domains such as knowledge of existing efforts, leadership support, implementation climate, health literacy of participants, and available resources. Changes in individual outcome parameters over time were analyzed using paired t-tests. Regression models were used to assess the association between participation in the intervention activities and changes in outcomes.

**Results:**

A total of nine organizations participated in the study. The organizational readiness assessment revealed an increase in management support, though a slight decline in the implementation climate was noted. A total of 171 participants (65% men; mean age = 51.9 years, SD = 9.9) were included in the baseline assessment, and 110 were included in the six-month follow-up. Approximately 50% of participants took part in the intervention activities. Positive trends over time were observed for food literacy and mental health literacy, but not for other outcomes. No clear pattern emerged in the relationship between participation in the intervention activities and changes in the outcome variables.

**Conclusion:**

This study revealed small improvements in proximal outcome variables indicating feasibility and potential impact. However, more research is necessary to determine the effectiveness of this novel problem-based learning intervention.

**Supplementary Information:**

The online version contains supplementary material available at 10.1186/s12889-025-25313-4.

## Introduction

In May 2024, 6.0% of the labor force in the European Union, approximately 13.2 million people, were unemployed [1]. Long-term unemployment, a subcategory of unemployment, is defined as being out of work and actively seeking employment for at least one year [2]. In Germany, about 40% of unemployed individuals are long-term unemployed [3]. Although unemployment rates have declined in many European countries over the past few decades [1], individuals experiencing long-term unemployment constitute a vulnerable population group with increased health risks, including mental illness, poor diet, physical inactivity, obesity, and higher mortality rates [4–8]. There is strong evidence of a reciprocal relationship between long-term unemployment and health, meaning that poor health increases the risk of long-term unemployment, and long-term unemployment, in turn, negatively affects health [4].

While it is unclear whether health literacy should be considered an independent social determinant of health, research has shown that it is associated with health-related behaviors and outcomes [9, 10]. Closely linked with education and language skills [11–14], health literacy has been defined in various ways. The European Health Literacy Consortium defines health literacy as the knowledge, motivation, and skills needed to access, understand, appraise, and apply health information to make decisions regarding healthcare, disease prevention, and health promotion in daily life [15]. However, health literacy should be understood not only as an individual trait or risk factor, but also as an asset of social entities, such as families, organizations, service providers, and communities [16, 17]. Health-literate social entities can empower for individuals to take action regarding their health and exercise control over their lives [18].

A population-based study from Denmark showed that unemployment is associated with inadequate health literacy [19]. Furthermore, preventative programs targeting individuals, such as health checkups, cancer screenings, and dental care are less successful at enrolling long-term unemployment individuals compared to employed individuals [6]. Several health promotion interventions that specifically target the long-term unemployed already exist, such as the JOBS program [20–22]. Most of these interventions aim to improve labor market integration and mental health, but very few have focused on health literacy as an outcome [23]. Furthermore, while recognizing the potential to reduce health disparities, a recent systematic review concluded that few health literacy interventions employ a clear theoretical framework, which limits both the interpretation and the transferability of these interventions [24, 25].

In Germany, individuals experiencing long-term unemployment can to work in nonprofit settings to regain employability and reintegrate into the labor market through programs such as Arbeitsgelegenheiten (1-Euro jobs, § 16 d SGB II) and Teilhabe am Arbeitsmarkt (labor market participation, § 16i SGB II). Within these nonprofit organizations for occupational rehabilitation, participants receive regular counseling from social workers. Thus, occupational rehabilitation offers an important opportunity to engage with people affected by long-term unemployment and to address their health literacy. In this study, we developed a framework for evidence-based interventions that address health literacy in occupational rehabilitation settings (FORESIGHT). We followed the steps of the Intervention Mapping (IM) approach to ensure that the intervention was theory- and evidence-based [26]. The study aimed to test the feasibility of the intervention elements under real-world conditions, as well as assess changes in health literacy and organizational readiness to address health literacy among participants. The research questions were:

- To what extent were the intervention elements implemented as planned?
- Are there changes in general health literacy and specific health literacies in the areas of nutrition, exercise, and mental health among participants?
- Are there changes in organizational readiness to address health literacy?

## Methods

### Study design

We used a pre-post study design to assess the feasibility of the intervention and the changes in the outcome variables. The aim was to implement the intervention in ten organizations working with individuals who had experienced long-term unemployment. Baseline assessments were conducted at both the individual and organizational levels between November 2022 and January 2023. Follow-up assessments were carried out six months later, from May to July 2023.

The sample size calculation was based on data from the German Health Update 2014/15 [27], which included the study’s primary outcome measure: the European Health Literacy Scale (HLS-EU-Q16). Analyses of survey data revealed mean scores of 12.4 and 13.2 respectively, among unemployed and employed individuals (standard deviation (SD) = 3.1). Based on these scores, we hypothesized that study participants would report an increase of 0.8 scale points in general health literacy, with an SD of 3.1. We estimated that sample size of 120 participants would be needed to detect such an increase using paired t-tests (α = 0.05, power = 0.80). Assuming a 20% loss to follow-up, we aimed for a sample size of 144. Data were collected via in-person interviews. All participants received written information about the study and provided informed consent for the use of their data. Study materials were available in multiple languages. Ethical approval was obtained from the Ethics Committee of the University of Bremen in November 2022 (reference number 2022–23).

### Setting and participants

The study was conducted in Bremen, a city in northwestern Germany with a population of approximately 570,000. Recruitment for the intervention took place within an umbrella organization, Verein arbeitsmarktpolitischer Dienstleister in Bremen (VaDiB e.V.), which comprises nonprofit organizations that work with long-term unemployed adults. Organizations received invitation letters to a meeting at which the study plan was presented. Social workers in the organizations served as the primary points of contact and completed the organizational readiness assessments, two except in two cases where operational management staff participated.

Individuals employed under the 1-Euro Job Act or the Labor Market Participation Act were eligible to participate in the study. This means that they had experienced long-term unemployment before being employed as part of an occupational rehabilitation measure. Organizations working in this field typically employ people in occupational rehabilitation measures as well as regular staff (e.g., administration staff). These regular employees of the participating organizations were excluded from the study.

With help of social workers, the study team distributed flyers and posters within the participating organizations to recruit study participants for the baseline and follow-up assessments. Social workers also invited individuals directly to participate. Potential participants registered with social workers, who placed them on a list for interviews. Participants received €10 as an incentive for completing each interview. Participation in the assessments was independent of participation in the intervention activities.

### Intervention

The intervention development followed the steps of the IM protocol [26] and aimed to involve key personnel (social workers) and the target group in the process. During the preplanning phase of the study, the research team liaised with one of the largest organizations in the occupational rehabilitation setting in Bremen (Groepelinger Recycling Initiative e.V.). The research team particularly worked with a social worker who also coordinated workplace health promotion activities across several organizations.

According to the first step of the IM protocol, a needs assessment was conducted. To this end, ten qualitative interviews with unemployed individuals were conducted, along with a scoping review focusing on health literacy needs. While the included literature primarily focused on mental health literacy, the qualitative interviews revealed that healthy nutrition and physical activity were also important topics. It was evident from the interviews that the intervention should include practical elements for establishing healthy routines. The interviewees identified the social workers at occupational rehabilitation organizations as a source of relevant health information and cues to action [28]. In a subsequent workshop with twelve unemployed individuals, the research team further explored competencies and lived experiences related to health literacy, well-being and health behaviors. The research team also discussed past health promotion efforts and practicalities related to the planned intervention in a workshop with five social workers from different organizations. Finally, another workshop was held with eleven unemployed persons to prioritize the intervention’s overall aims. As the second and the third steps of IM, the research team drafted a logic model of change and selected an underlying theory. The model of change was based on the insights from the needs assessment and two additional reviews. In these reviews in which the research team identified predictors of health literacy and effective intervention strategies [29, 30]. The intervention was theoretically grounded in the concept of health literacy, whichencompasses finding, understanding, appraising, and applying health information (as defined by Sørensen et al. [15]), and the COM-B model (Capabilities, Opportunities, Motivation – Behavior; [31]). Following the IM protocol, the research team defined performance objectives for each thematic area of the intervention: nutrition, physical activity, mental health. Next, matrices of change were created along the domains of the health literacy concept and the COM-B model. The research team presented the logic model of change to eight social workers and discussed ideas for practical strategies to deliver the intervention. During the session the social workers suggested that intervention should have regular elements (e.g., weekly meetings) but should also accommodate differences in needs and preferences across organizations.

The fourth step in the IM protocol involved producing the intervention program. To this end, intervention materials were drafted and pretested in one organization. The refined intervention program was then presented to social workers and management staff from 14 organizations that worked with long-term unemployed individuals in Bremen. The social workers were the main contact points for the research team and acted as change agents within their organizations. For this purpose, they received theoretical input on the concept of organizational health literacy and how to address it.

The last two steps of IM involved the implementation and evaluation of the intervention. This paper presents the results of these steps.

Overall, the intervention program addressed health literacy in the areas of nutrition, physical activity, and mental health. It combined theoretical sessions using a problem-based learning (PBL) approach with practical sessions.

During the PBL sessions, participants were presented with cases describing health problem or work-related issues to solve. PBL was deemed particularly suitable because it aligns with the idea that health literacy is a generative practice rather than a fixed set of knowledge and skills [32]. A review conducted as part of the IM also found PBL interventions to be particularly effective [30]. The sessions were designed to motivate participants to engage with health literacy by presenting relatable cases that did not require disclosing personal health issues. Active problem-solving was intended to foster skills in dealing with health-related information. Unlike traditional PBL approaches in medical education, this intervention included more guidance and session-specific tasks and were written in simple language avoiding technical terms. Each of the three case stories extended over four weekly one-hour sessions, which were usually led by social workers within the organization. In exceptional cases, a community health educator and a research team member acted as facilitators. The facilitators received detailed session-by-session guidelines and attended a Q&A session before implementation.

The practical sessions included cooking classes or smoothie days focused on nutrition, physical activity classes, and relaxation techniques to promote mental health. These sessions aimed to bridge the gap between capabilities and opportunities by addressing the need for improvement in applying health information, i.e., putting knowledge into action, which was identified during the needs assessment. To reduce barriers to participation, the PBL and practical sessions were conducted at the workplace during work hours and facilitated by either external experts or social workers from the participating organizations.

The intervention program allowed organizations to choose among six activities based on their needs and preferences as recommended by the social workers during the IM process. A description of the activities is provided in Table 1.

**Table 1:** Description of the six intervention activities

Area	Activity
Nutrition	Problem-based learning: At the end of these units, participants should be able to (1) describe what is meant by healthy eating and what the key recommendations of the German Nutrition Society are; (2) identify symptoms of a coronary heart disease, (3) recognize the benefits of using multiple sources of information, (4) appraise the trustworthiness of sources of health information between good and bad sources of information, (4) to better assess their own eating habits by keeping a food diary
	Practical activities: Monthly cooking classes on cooking using regional and seasonal unprocessed foods, comparing prices of processed and unprocessed foods, information on sugar content in processed foods, reading food labels, meatless food alternatives. Monthly Smoothie days: food ingredients, exploring taste sensations, preparation according to a recipe, trying out new food combinations
Physical activity	Problem-based learning: Key learning objectives: At the end of these units, participants should be able to (1) identify sources of information on the subject of exercise and one-sided physical strain in everyday working life, (2) understand physical problems that can be associated with a lack of exercise or one-sided physical strain in their everyday life, (3) assess the needs and opportunities for health-promoting exercise programs in their company, (4) design a suitable program for their own company
	Practical activities: Weekly back fitness courses, Qi Gong, or repair shop gymnastics
Mental health	Problem-based learning: Key learning objectives: At the end of these units, participants should be able to (1) recognize and understand depression as a serious illness, (2) recognize the symptoms of depression (3) seek and find support for mental health problems (4) identify sources of information on mental health and search for this information, (5) critically question the trustworthiness of sources used (6) better assess whether they or someone around them needs mental health support
	Practical activities: Qi Gong, breathing techniques, dream journey

### Measures

In alignment with the study’s overall aim and the specific topics addressed in the intervention activities, several health literacy scales were selected for the pre- and post-assessments. At the individual level, general health literacy, measured by the HLS-EU-Q16, was the primary outcome variable [33]. The scale contains 16 statements in which participants rate how easy or difficult it is to find, understand, appraise, or use health information for healthcare, disease prevention, and health promotion (internal consistency α = 0.82 at baseline and 0.83 at follow-up). A total score was calculated by dichotomizing the four response options (very easy/fairly easy = 1; fairly difficult/very difficult = 0) and summing the scores (possible range 0–16). Participants with more than two missing values were excluded from the analysis, as recommended by the scale developers [34].

Additionally, the Newest Vital Sign test was used to assess participants' functional health literacy [35]. In this test, participants are given a food label from an ice cream container and asked six questions requiring basic literacy and numeracy skills. According to the test instructions, 0–1 correct answers indicate limited health literacy, 2–3 suggest possible limited health literacy, and 4–6 reflect adequate health literacy [35].

Due to the emphasis on nutrition, physical activity, and mental health, additional health literacy scales specific to these topics were included as proximal outcome variables. Food literacy was assessed using six items from the Food Literacy Scale that focus on food label use, daily food planning, and healthy budgeting [36]. Responses ranged from 1 (no, never) to 5 (yes, always) (internal consistency α = 0.75 at baseline and 0.83 at follow-up). A summary score was calculated for the analysis (possible range 6–30). Physical activity literacy was measured using the control competence subscale from the physical activity-related health competence scale [37], addressing understanding of body signals and knowledge of appropriate exercises for physical fitness. This scale includes six items with four response options (1 = strongly disagree; 4 = strongly agree; internal consistency α = 0.78 at baseline and 0.71 at follow-up; possible range 1–4). Mental health literacy was assessed using the Mental Health Knowledge Schedule (MAKS) [38], which includes 12 items addressing stereotypes, attitudes, and knowledge about mental illnesses. Responses ranged from 1 (strongly disagree) to 5 (strongly agree; internal consistency α = 0.54 at baseline and 0.56 at follow-up; possible range 12–60).

Sociodemographic information, including age, gender, country of birth, education, and self-rated health, was collected during the baseline interview. In the follow-up survey, participants were also asked about their participation in intervention activities and their satisfaction with them (1 = not satisfied at all; 5 = very satisfied). Non-participants or partial participants were asked to provide reasons for their lack of full participation.

In addition to individual-level assessments, a structural evaluation was carried out. An assessment of the contextual preconditions is important because they can influence the implementation success [39]. Furthermore, the social workers were tasked with initiating changes within their organization towards to promote health literacy beyond the defined intervention activities. To evaluate the organizational readiness for change, baseline and follow-up key informant interviews were conducted with social workers and, in some cases, operational management. The assessment was based on the community readiness assessment [40]. The interview consisted of approximately 40 questions addressing five readiness dimensions: (1) knowledge among potential participants about existing workplace health (literacy) promotion efforts; (2) leadership support from operational management; (3) implementation climate among potential participants (i.e., their willingness to engage in workplace health (literacy) promotion); (4) health literacy of potential participants; and (5) available resources. In the original assessment, dimension 4 addresses the target population's knowledge about the health issue. In our assessment, this dimension was modified to evaluate potential participants' general health literacy using the HLS-EU-Q6 [41]. Answers in each dimension were scored on a scale ranging from 1 (no awareness) to 9 (professionalization) according to the community readiness manual [40]. According to the community readiness model, readiness scores should be ≥ 4 (preplanning phase) before implementing an intervention. The follow-up interview included additional questions about the implementing intervention activities, satisfaction with these activities (1 = very bad; 5 = very good), and the number of participants.

English versions of the questionnaire and interview topic guides can be found in the supplementary file.

### Statistical analysis

Descriptive statistics were used to analyze the participant characteristics, the organizational readiness assessment and the implementation variables. Paired t-tests were used to analyze changes in the outcome variables. For the ordinal outcome variable (functional health literacy) the paired Wilcoxon signed-rank test was used. Additional analyses explored participation in the intervention activities and change in the outcome variables using linear and ordinal regression analyses. Furthermore, gender-stratified analyses were carried out to explore differences between women and men. There were a very few missing values in the outcome variables (max. 2.7%). Pairwise deletion was used to handle these missing values. All statistical analyses were performed using Stata 16 (Stata Corp., College Station, TX, USA).

## Results

A total of ten nonprofit organizations were recruited for the study. Nine of these organizations ultimately implemented intervention activities and participated in the pre- and post-assessments. These organizations operated in various sectors, including gardening, recycling and repair shops, gastronomy, and charity shops. The organizations varied in size. One organization employed fewer than ten potential participants, three organizations employed ten to thirty potential participants, and five organizations employed more than thirty potential participants.

### Structural evaluation

**Fig. 1:**
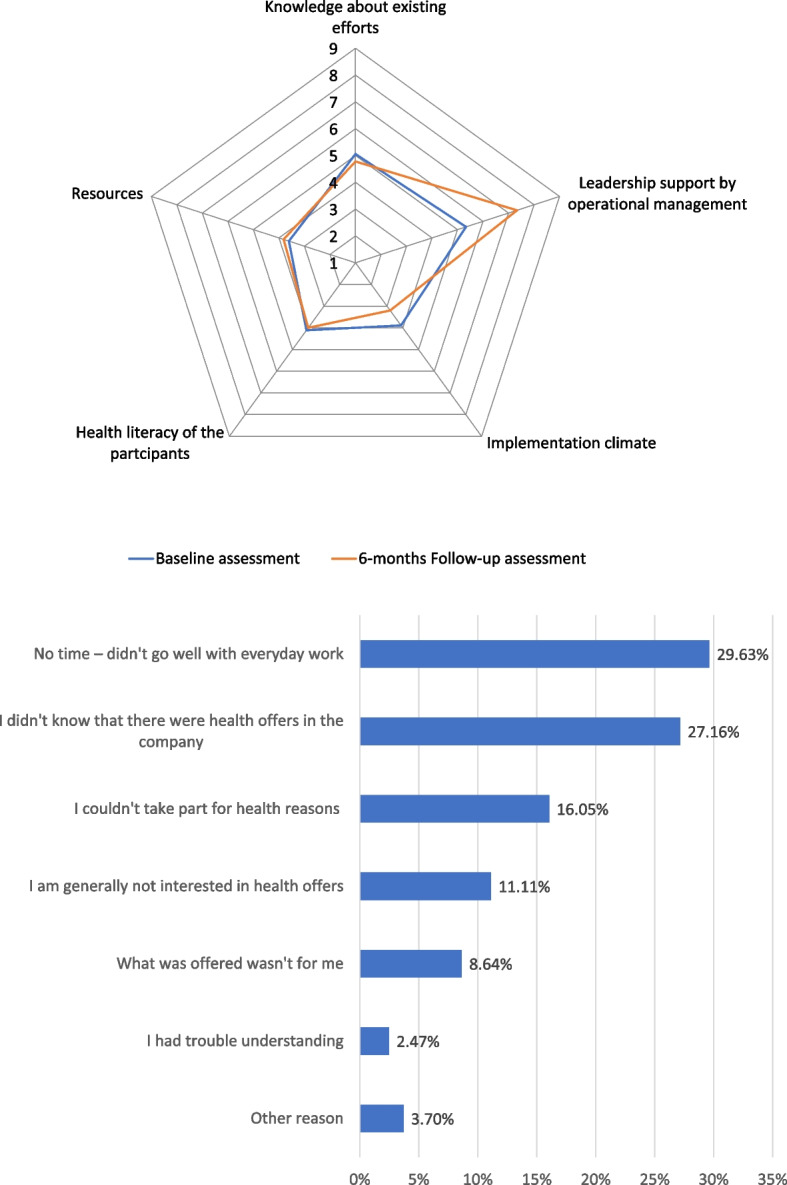
Comparison of the baseline and follow-up organizational readiness scores across nine occupational rehabilitation workplaces (*n* = 21 key informant interviews from nine organizations)

A total of 21 interviews were conducted for the baseline and follow-up readiness assessments (four men and 17 women respondents; age range 35–64 years). In two organizations, both the social worker and the operational management participated in the assessment. In these cases, the scores from the two interviews were averaged. In all other cases, the social workers were the sole key informants per organization. A comparison of the baseline and follow-up readiness assessments showed a slight increase in the overall mean readiness score, rising from 4.4 (SD = 1.1) to 4.6 (SD = 1.1). Figure 1 shows an increase in leadership support from operational management, from 5.3 (SD = 2.9) to 7.3 (SD = 0.8). This means that organizational readiness moved from the preparation phase to the stabilization phase in this domain. Conversely, the implementation climate decreased slightly from 3.9 (SD = 1.8) to 3.2 (SD = 1.3). The three remaining domains remained stable. On average, the organizations implemented 2.8 (SD = 1.5, range 1–5) of the six intervention activities.

### Implementation of the intervention activities

The key informants provided information on how many participants attended the intervention activities. These numbers differed from those in the pre-post comparison at the individual level because not all attendees participated in the study. Overall, about 80 people participated in the PBL activities. Group sizes ranged from two to eight (mean = 5.3, SD = 1.8). Approximately 31 people attended the cooking classes, and an additional 118 people were reached through Smoothie Days. According to key informants, physical activity classes had approximately 180 participants. It was challenging to assess participation in the mental health promotion activities (e.g., relaxation techniques), because they were often associated with physical activity, as in the case of Qi Gong. According to key informants, only four to five people participated in other relaxation exercises, such as breathing techniques and dream journey.

Table 2 shows the frequency of activity implementation and the satisfaction levels of key informants and participants with these activities. The PBL case on healthy nutrition and the practical activities related to physical activity and nutrition were the most popular. On average, the key informants and participants rated their satisfaction positively.

**Table 2:** Implementation of the six intervention activities

Area	Content	Number of implementing organizations	Satisfaction ratings by key informants Mean (SD) (*n* = 3–10)	Satisfaction ratings by participants Mean (SD) (*n* = 8–28)
Nutrition	PBL	8	4.2 (0.6)	4.3 (0.7)
	Practical activities	5	4.4 (0.5)	4.2 (0.8)
Physical activity	PBL	3	4.2 (1.0)	4.1 (0.8)
	Practical activities	5	4.4 (0.5)	4.4 (0.6)
Mental health	PBL	3	4.8 (0.5)	3.9 (1.3)
	Practical activities	3	4.3 (0.6)	-

*PBL* Problem-based learning, *SD* Standard deviation

### Individual evaluation

A total of 171 individuals with a history of long-term unemployment participated in the baseline survey (T0). Of these, 110 of these also participated in the six-month follow-up survey (T1), resulting in a loss-to-follow-up of 36%. About two-thirds of the participants were men, and about one-fourth were foreign-born, reflecting the composition of the workforces in the participating organizations. More than 50% of the participants rated their health as fair or poor. The main reasons for not participating in the second survey were termination of employment in the organization and long-term illness. As shown in Table 3, the characteristics of baseline study sample and follow-up completers were nearly identical, indicating no selection bias.

**Table 3:** Baseline characteristics of the whole study sample (T0) in comparison to the follow-up completers (T0/T1)

	n	T0	n	T0/T1
		M/%		M/%
Mean Age	171	51.9 (SD 9.9, range 27–73)	110	51.0 (SD 10.5, range 27–67)
Gender				
Man	112	65.5%	72	65.4%
Woman	59	34.5%	38	34.6%
Diverse	-	0%	-	0%
Country of birth				
Germany	121	70.8%	82	74.6%
Other country	50	29.2%	28	25.4%
Highest educational degree				
No degree yet/special school	22	12.9%	16	14.6%
Secondary school leaving certificate	135	79.0%	85	77.3%
University entrance qualification	12	7.0%	7	6.4%
Missing	2	1.2%	2	1.8%
Self-rated health				
Excellent	5	2.9%	4	3.6%
Very good	14	8.2%	9	8.2%
Good	63	36.8%	40	36.4%
Fair	69	40.4%	45	40.9%
Poor	20	11.7%	12	10.9%

**Fig. 2:**
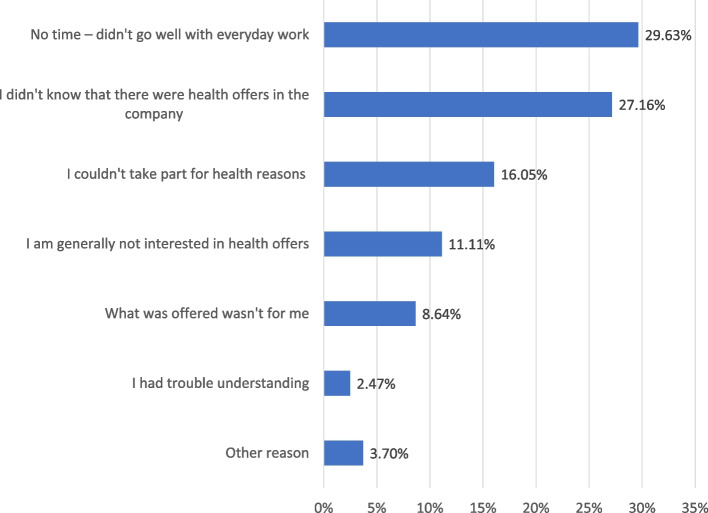
Reasons for partial or non-participation in the intervention activities (*n* = 81)

Among the respondents in to follow-up survey, 53% reported participating in at least one intervention activity. Figure 2 shows the reasons for not fully participating in the intervention activities, as reported by the survey respondents. Lack of time during work and unfamiliarity with the activities were the most frequently mentioned reasons.

An analysis of changes in the outcome measures for the entire sample showed significant improvements in food literacy and mental health literacy (Table 4). However, no changes were observed in general health literacy, functional health literacy, or physical activity literacy. A stratified analysis by gender did not reveal major differences between men and women (Table S1 in the supplement). Exploring the association between the level of participation in the intervention activities did not reveal a clear dose–response relationship. However, three out of five regression estimates were highest when both PBL sessions and practical activities were attended (Table S2). Due to the small number of cases in some cells—for example, only six participants attended both nutrition-related PBL sessions and nutrition-related practical activities—the statistical precision of this analysis was limited.

**Table 4:** Change over time in health literacy outcomes (paired t-tests, *n* = 110)

Outcome parameters	Baseline Means (SD, range)	6-Month Follow-up Means (SD, range)	Mean Difference (95% CI)
General health literacy	11.4 (3.5, 2–16)	11.2 (3.2, 3–16)	-.14 (-.64;.36)
Functional health literacy	Limited = 37.3% Possible limited = 29.1% Adequate = 33.6%	Limited = 43.6% Possible limited = 22.7% Adequate = 33.6%	-§
Food literacy	15.3 (5.6, 6–29)	16.1 (6.1, 6–30)	.80 (.02; 1.58)
Physical activity literacy	3.02 (.55, 1.2–4.0)	3.00 (.51, 1.7–4.0)	-.02 (-.10;.05)
Mental health literacy	40.9 (6.0, 12–60)	42.3 (6.7, 12–58)	1.39 (.37; 2.41)

^§^Wilcoxon signed rank test: no significant change

## Discussion

In this study, we developed and tested a health literacy intervention for long-term unemployed individuals in a real-world context. The intervention was designed using the steps of the IM protocol and involved both the target group and stakeholders in the development process. The approach allowed organizations to select and implement activities the most appropriate activities for their specific contexts. The intervention incorporated motivational, educational and practical approaches, which have recently been demonstrated to result in health improvements among middle-aged adults [42]. Moreover, it is one of a limited number of interventions that is focused on health literacy as an outcome [23]. Results from the baseline and follow-up assessments in this study showed small improvements in two proximal outcomes (food literacy and mental health literacy) indicating feasibility and potential impact. However, there was no significant change in the primary outcome of general health literacy. Additionally, there was an increase in leadership support from operational management, but a slight decline in the implementation climate among potential participants.

The data from our study highlight the urgent need to address health literacy and health issues among the long-term unemployed. Approximately 40% of participants had limited functional health literacy, which is four times higher than in the general German population [43]. Additionally, since health problems are a major cause of long-term unemployment, only about 50% of participants rated their health as good to excellent. This figure is roughly 20 percentage points lower than that of the general German population [44]. As expected, health problems were among the main reasons for loss to follow-up and low participation in intervention activities in our study.

To keep the threshold for adopting of the intervention low, key personnel and the target group within the organization could choose from different intervention activities based on their needs and preferences. However, incomplete implementation of the intervention may explain why no changes in general health literacy were observed [45]. The fact that positive trends were found in the area where most intervention activities were concentrated (nutrition) support this explanation. Another possible reason for the lack of observed changes in general health literacy could be the nature of the HLS-EU-Q16 scale. Although it is widely used, the scale has been criticized for some of its items [46]. An additional analysis of our study data showed no correlation between the self-reported HLS-EU-Q16 scores and test-based functional health literacy [47]. Additionally, a cognitive interview study revealed that many participants did not fully understand or interpret several HLS-EU-Q16 items as intended [48]. The lack of changes in functional health literacy may also be attributed to the fact that it relies on basic literacy and numeracy skills, which may require additional education to improve.

Despite involving members of the target group in developing the intervention and offering activities during working hours, low participation was a critical issue in our study. According to our structural evaluation, the implementation climate, defined as the motivation and willingness of potential participants to engage with the intervention, was low at baseline and was rated even lower in the follow-up assessment. The Consolidated Framework for Implementation Research (CFIR) suggests that the implementation climate plays a crucial mediating role between management support and effectiveness [49, 50]. Non-participants cited lack of time and unawareness of the intervention activities as the main reasons for not participating, indicating that employers did not actively make time for all participants to join the activities. In discussions with social workers from the implementing organizations, the social workers speculated that these reasons might have been excuses to avoid participation, or that potential participants feared being judged by their coworkers. In any case, effectively communicating the intervention activities to potential participants and fostering a positive implementation climate were significant challenges. Low participation was also an issue in a recent nationwide replication study of the JOBS program in Germany, in which only 94 of the targeted 1,500 unemployed individuals participated [51, 52].

This study has several strengths and limitations. The strengths include the participatory and theory-based development of the intervention, testing it in a real-world setting, and conducting a comprehensive evaluation that addressed both the individual and structural levels. A major limitation is the lack of a control group, which prevents us from assessing the intervention's effectiveness. Additionally, while more participants were recruited at baseline than planned, the number of participants lost to follow-up was higher than anticipated. Overall, the sample size was too small, and participation in the intervention activities was too low to establish dose–response relationships. Furthermore, the primary outcome measure, the HLS-EU-Q16, may not be an appropriate instrument for assessing health literacy in this population.

## Conclusion

This study demonstrated the feasibility of the intervention and its potential impact by revealing small improvements in food literacy and mental health literacy among long-term unemployed individuals, as well as increased management support for promoting health literacy within participating organizations. While occupational rehabilitation organizations appear to be appropriate settings for addressing health literacy, achieving high levels of participation among the target group and fully implementing intervention activities remain significant challenges.

Therefore, practitioners and researchers should further explore strategies to improve the implementation climate among long-term unemployed individuals and engage them more effectively in health literacy promotion activities. Despite the positive trends in some proximal outcomes, a more rigorous research design is needed to definitively demonstrate the effectiveness of our intervention approach.

## Supplementary Information

Supplementary Material 1

## Data Availability

The data that support the findings of this study are available from the corresponding author on reasonable request.
