# Supplementary material for: Addressing health literacy among long-term unemployed persons: the FORESIGHT intervention study
Source: BMC Public Health. 2025 Nov 17;25:3967. doi: 10.1186/s12889-025-25313-4 (PMC12621407; doi:10.1186/s12889-025-25313-4)
Supplement: Supplementary file 1 — Supplementary Material 1 [file 12889_2025_25313_MOESM1_ESM.docx]

**Table S1.** Gender stratified analyses of change over time in health literacy outcomes

| **Outcome parameters** | **Baseline**  **scale means (SD)** | **6-Month Follow-up**  **scale means (SD)** | **Difference (95% CI)** |
| --- | --- | --- | --- |
| Men (n=72) | | | |
| General health literacy | 11.3 (3.8) | 10.9 (3.5) | -.35  (-.96; .25) |
| Functional health literacy | Limited=45.8%  Possible limited=26.4%  Adequate =27.8% | Limited=51.4%  Possible limited=18.1%  Adequate =30.6% | -* |
| Food literacy | 14.9 (5.7) | 16.0 (6.5) | 1.05  (.00; 2.11) |
| Physical activity literacy | 3.02 (.59) | 3.03 (.52) | .01  (-.08; .11) |
| Mental health literacy | 40.9 (6.1) | 42.3 (5.7) | 1.40  (.13; 2.68) |
| Women (n = 38) | | | |
| General health literacy | 11.5 (2.9) | 11.8 (2.7) | .27  (-.64; 1.17) |
| Functional health literacy | Limited=21.1%  Possible limited=34.2%  Adequate =44.7% | Limited=28.9%  Possible limited=31.6%  Adequate =39.5% | -* |
| Food literacy | 16.2 (5.4) | 16.5 (5.2) | .30  (-.78; 1.38) |
| Physical activity literacy | 3.04 (.48) | 2.94 (.48) | -.10  (-.23; .03) |
| Mental health literacy | 41.0 (7.7) | 42.3 (6.6) | 1.36  (-.42; 3.15) |

* Wilcoxon signed rank test: no significant change

**Table S2.** Association between participation in intervention activities and health literacy outcomes at 6-month follow-up (linear and ordinal regressions)

|  | **General health literacy** | | **Functional health literacy*** | | **Food literacy** | | **Physical activity literacy** | | **Mental health literacy** | |
| --- | --- | --- | --- | --- | --- | --- | --- | --- | --- | --- |
|  | Regression coefficient (95% CI) | | | | | | | | | |
| No participation | n=53 | Ref. | n=53 | Ref. | n=65 | Ref. | n=53 | Ref. | n=53 | Ref. |
| Only practical activities | n=13 | -.43  (-1.62; .76) | n=13 | 1.21  (.42; 3.53) | n=18 | -.87  (-3.03; 1.28) | n=13 | -.10  (-.28; .07) | n=13 | 1.89  (-.38; 4.15) |
| Only PBL | n=23 | .61  (-.83; 2.05) | n=23 | 1.96  (.51; 7.46) | n=21 | .35  (-1.64; 2.34) | n=23 | -.16  (-.38; .06) | n=23 | 1.68  (-1.16; 4.52) |
| Both | n=21 | -.35  (-1.54; .85) | n=21 | 1.99  (.67; 5.93) | n=6 | 2.42  (-.95; 5.78) | n=21 | .16  (-.03; .34) | n=21 | .75  (-1.70; 3.21) |

PBL problem-based learning. All models were adjusted for baseline scores in the outcome variable.

* Ordinal regression, the coefficient is odds ratios; all other estimates are unstandardized linear regression coefficients.

# Baseline Questionnaire


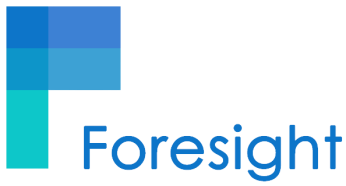


Framework for evidence-based interventions to promote health literacy in the job-reintegration setting

Survey on dealing with health information

| **Variable name** |  | |  |
| --- | --- | --- | --- |
| PID | Respondent’s ID | __\|__\|__\|__\|__\|__\| |  |
| Int1 | Interviewer ID | __\|__\|__ |  |
| Int2 | Institution | ___________________________________ |  |
| Int3 | Interview language | __ _1_ German  __ _2_ English  __ _3_ Russian  __ _4_ Arabic |  |
| Int4 | Date | __\|__. __\|__. __\|__\|__\|__  DD MM YYYY |  |
| Int5 | Time (Start) | __\|__. __\|__  Hour Min. |  |

**Thank you once again for agreeing to participate in this interview. I’m going to ask a you few questions concerning your health, how you deal with health information, as well as** **your living conditions.**

**If you don’t have any questions, I will start with the first questions.**

| **General Information** |
| --- |

**To begin with I’d like to ask you some general questions.**

**Age**

**Q1 How old are you?**

__|__ Years __ _998_ [Don’t know] __ _999_ [Not specified]

**Gender**

**Q2 What is your gender?**

__ _1_ Male

__ _2_ Female

__ _3_ Other

__ _8_ [Don’t know]

__ _9_ [Not specified]

**Career**

**Q3 How long have you been working at this institution?**

__|__ Years __|__ Months

**Self-rated health**

**Q4 In general, would you say that your health is…**

*INT: Present show card 1*

__ _1_ Excellent

__ _2_ Very good

__ _3_ Good

__ _4_ Fair

__ _5_ Poor

__ _8_ [Don’t know]

__ _9_ [Not specified]

| **Dealing with health information** |
| --- |

**Now we are going to focus on how you generally deal with health information.**

**General health literacy**

**Q5 On a scale from very easy to very difficult, how easy would you say it is to…**

*INT: Present show card 2*

- Please tick or cross one box in each row!

|  |  | Very easy | Fairly easy | Fairly  difficult | Very  difficult | Don’t know/refusal |
| --- | --- | --- | --- | --- | --- | --- |
| **Q5a** | ...find information on treatments of illnesses that concern you? | __ _1_ | __ _2_ | __ _3_ | __ _4_ | __ _8_ |
| **Q5b** | ...find out where to get professional help when you are ill? (Doctor, pharmacist, psychologist) | __ _1_ | __ _2_ | __ _3_ | __ _4_ | __ _8_ |
| **Q5c** | ...understand what your doctor says to you? | __ _1_ | __ _2_ | __ _3_ | __ _4_ | __ _8_ |
| **Q5d** | ...understand your doctor’s or pharmacist’s instructions on how to take a prescribed medicine? | __ _1_ | __ _2_ | __ _3_ | __ _4_ | __ _8_ |
| **Q5e** | ...judge when you may need a second opinion from another doctor? | __ _1_ | __ _2_ | __ _3_ | __ _4_ | __ _8_ |
| **Q5f** | ... use the information the doctor gives you to make decisions about your illness? | __ _1_ | __ _2_ | __ _3_ | __ _4_ | __ _8_ |
| **Q5g** | ...follow instructions from your doctor or pharmacist? | __ _1_ | __ _2_ | __ _3_ | __ _4_ | __ _8_ |
| **Q5h** | ... find information about support services for mental health problems such as stress or depression? | __ _1_ | __ _2_ | __ _3_ | __ _4_ | __ _8_ |
| **Q5i** | ...understand health warnings about behaviour such as smoking, low physical activity and drinking too much? | __ _1_ | __ _2_ | __ _3_ | __ _4_ | __ _8_ |
| **Q5j** | ...understand why you need health screenings? (Cancer screening, blood sugar tests, blood pressure) | __ _1_ | __ _2_ | __ _3_ | __ _4_ | __ _8_ |
|  |  | Very easy | Fairly easy | Fairly  difficult | Very  difficult | Don’t know/refusal |
| **Q5k** | … assess if the information on health risks in the media is reliable? (TV, Internet or other media) | __ _1_ | __ _2_ | __ _3_ | __ _4_ | __ _8_ |
| **Q5l** | ...decide how you can protect yourself from illnesses based on the information from the media? (Newspapers, leaflets, Internet or other media) | __ _1_ | __ _2_ | __ _3_ | __ _4_ | __ _8_ |
| **Q5m** | ... find information about activities/behaviours that are good for your mental well-being? (Meditation, physical activity, going for a walk, Pilates etc.) | __ _1_ | __ _2_ | __ _3_ | __ _4_ | __ _8_ |
| **Q5n** | ...understand health advice from family members or friends? | __ _1_ | __ _2_ | __ _3_ | __ _4_ | __ _8_ |
| **Q5o** | ...understand information in the media on how to get healthier? (Internet, newspapers, magazines) | __ _1_ | __ _2_ | __ _3_ | __ _4_ | __ _8_ |
| **Q5p** | ...assess which everyday behaviour is related to your health? (Drinking and eating habits, physical activity etc.) | __ _1_ | __ _2_ | __ _3_ | __ _4_ | __ _8_ |

**Nutrition-related Health Literacy**

**I will now ask you a few questions about nutrition.**

*INT: Present show card 3 for all questions in this block.*

*Also present show card 4 for Q6d,*

*and show card 5 for Q6e*

|  |  | Yes,  always | Yes, usually | Sometimes yes, sometimes no | No, usually not | No, never | Don’t know/  refusal |
| --- | --- | --- | --- | --- | --- | --- | --- |
| **Q6a** | If you have something to eat, do you consider what you will eat later that day? | __ _1_ | __ _2_ | __ _3_ | __ _4_ | __ _5_ | __ _8_ |
| **Q6b** | If you have something to eat, do you consider what you ate earlier that day? | __ _1_ | __ _2_ | __ _3_ | __ _4_ | __ _5_ | __ _8_ |
| **Q6c** | Do you purchase healthy food, even if you have limited money?  For example, vegetables, fruit, or whole grain products. | __ _1_ | __ _2_ | __ _3_ | __ _4_ | __ _5_ | __ _8_ |
| **Q6d** | Do you check the nutritional labels of products for calories, fat, sugar or salt content? | __ _1_ | __ _2_ | __ _3_ | __ _4_ | __ _5_ | __ _8_ |
| **Q6e** | Do you pay attention to the Nutri-Score when shopping? | __ _1_ | __ _2_ | __ _3_ | __ _4_ | __ _5_ | __ _8_ |
| **Q6f** | Do you compare the calories, fat, sugar or salt content of different products? | __ _1_ | __ _2_ | __ _3_ | __ _4_ | __ _5_ | __ _8_ |

**In the next section, I would like to hear your opinion.**

- Please tick or cross one box in each row!

|  |  | Right | Wrong | Don’t know/  refusal |
| --- | --- | --- | --- | --- |
| **Q7a** | If you have eaten high-fat foods, you can reverse the effects by eating apples | __ _1_ | __ _2_ | __ _8_ |
| **Q7b** | A healthy meal should consist of half meat, a quarter vegetables and a quarter side dishes | __ _1_ | __ _2_ | __ _8_ |
| **Q7c** | Fat is always bad for your health; you should therefore avoid it as much as possible | __ _1_ | __ _2_ | __ _8_ |
| **Q7d** | A balanced diet implies eating all foods in the same amounts | __ _1_ | __ _2_ | __ _8_ |
| **Q7e** | To eat healthily, you should eat less fat. Whether you also eat more fruit and vegetables does not matter | __ _1_ | __ _2_ | __ _8_ |
| **Q7f** | For a healthy nutrition, dairy products should be consumed in the same amounts as fruit and vegetables | __ _1_ | __ _2_ | __ _8_ |
| **Q7g** | Brown sugar is much healthier than white sugar | __ _1_ | __ _2_ | __ _8_ |

**Experts give recommendations on what to eat regularly and how much of it.**

**Q8 According to experts, at least how many portions of fruit and vegetables should people eat daily (e.g., according to the World Health Organisation - WHO)? (A portion could for example be an apple or a handful of chopped carrots)**

(Cross or tick one box)

| 2 | 3 | 4 | 5 or more | Not sure |
| --- | --- | --- | --- | --- |
| __ _1_ | __ _2_ | __ _3_ | __ _4_ | __ _5_ |

**Newest Vital Sign**

***Interviewer instructions***

- There is no maximum time allowed to answer the questions. The average time needed to complete all 6 questions is about 3 minutes. However, if an interviewee is still struggling with the first or second question after 2 or 3 minutes, the likelihood is that the interviewee has limited literacy and you can stop the assessment.
- **Ask the questions in sequence.** Continue even if the interviewee gets the first few questions wrong. However, **if question 9e is answered incorrectly, do not ask question 9f.**
- **You can stop asking questions if an interviewee gets the first four correct.** With four correct responses, the patient almost certainly has adequate literacy.
- **Do not prompt interviewees who are unable to answer a question.** Prompting may jeopardize the accuracy of the test. Just say, “Well, then let’s go on to the next question.”
- **Do not show the score sheet to interviewees.** If they ask to see it, tell them “I can’t show it to you because it contains the answers, and showing you the answers spoils the whole point of asking you the questions.”
- **Do not tell interviewees if they have answered correctly or incorrectly.** If interviewees ask, say something like: “I can’t show you the answers till you are finished, but for now you are doing fine. Now let’s go on to the next question.”
- Tailor the explanation and the questions to the interviewee’s language level to enhance understanding

**We want health information to be worded in a way that is easy to understand. Would you help us by taking a look at the following information? This is about health information on food packaging.**

***I would then ask you a few more questions*.** *INT: Present show card 6*

**Imagine you have just bought a container of ice cream and you find this nutritional table on it. Please take a close look at this table and I will ask you a few questions about it. It doesn’t matter if you can’t answer all the questions. Some of them are difficult on purpose. Take as long as you need to answer each question.**

| **Q9a** | If you eat the entire container, how many calories will you eat?  *Correct answer: 1000 calories* |  |
| --- | --- | --- |
| **Q9b** | If you are allowed to eat 60 grams of carbohydrates as a snack, what is the maximum amount of ice cream you can have?  *Correct answer: 200ml or 2 portions (2 cups)* |  |
| **Q9c** | Imagine your doctor advises you to reduce the amount of saturated fat in your diet. You usually have 42 g of saturated fat each day, which includes one serving of ice cream. If you stop eating ice cream, how many grams of saturated fat would you be consuming each day?  *Correct answer: 33g* |  |
| **Q9d** | If you usually eat 2,500 calories in a day, what percentage of your daily value of calories will you be eating if you eat one serving?  *Correct answer: 10%* |  |
| **Q9e** | **Pretend that you are allergic to the following substances: penicillin, peanuts, latex gloves, and bee stings.**  Is it safe for you to eat this ice cream?  *Correct answer: No* |  |
| **Q9f** | Why not?  *Correct answer: Because the ice cream has peanut oil* |  |

**Physical activity-related health literacy**

**We now come to the topic of physical activity. The next statements are on how well you can assess your body. I will read out each statement to you and you tell me whether you fully agree, rather agree, rather disagree, or fully disagree with it.**

*INT: Present show card 7*

**Q10**

- Please tick or cross one box in each row!

|  |  | Fully agree | Rather agree | Rather disagree | Fully disagree | Don’t know/ Refused |
| --- | --- | --- | --- | --- | --- | --- |
| **Q10a** | I know what to look out for when it comes to my body so that I don't strain myself too much or too little. | __ _1_ | __ _2_ | __ _3_ | __ _4_ | __ _8_ |
| **Q10b** | I can use my body signals (pulse, breathing speed) very well to assess and regulate the level of physical strain | __ _1_ | __ _2_ | __ _3_ | __ _4_ | __ _8_ |
| **Q10c** | If I want to enhance my health by strengthening trunk muscles (back, abdomen), I am confident that I know the right exercises to do | __ _1_ | __ _2_ | __ _3_ | __ _4_ | __ _8_ |
| **Q10d** | If my muscles are tensed up, I know exactly how to counter this through physical activity | __ _1_ | __ _2_ | __ _3_ | __ _4_ | __ _8_ |
| **Q10e** | I am able to adjust training intensity well to my physical condition | __ _1_ | __ _2_ | __ _3_ | __ _4_ | __ _8_ |
| **Q10f** | I know how to use physical training to improve my endurance in the best possible way | __ _1_ | __ _2_ | __ _3_ | __ _4_ | __ _8_ |

**Experts, for example, from the World health organisation (WHO) give recommendations on how often adults (up to 65 years) should ideally be physically active in their everyday life.**

**Q11a. How many minutes or hours per week should adults engage in at least moderate-intensity physical activity or exercise?**

| 30 minutes | 1 hour | 1 ½ hours | 2 ½ hours | Not sure |
| --- | --- | --- | --- | --- |
| __ _1_ | __ _2_ | __ _3_ | __ _4_ | __ _5_ |

**Q11b. This basic recommendation can also be achieved by a shorter duration of high-intensity sports or exercise, or by a combination of the two. By how many minutes or hours (per week) of sport and/or exercise with higher intensity can the basic recommendation be achieved?**

| 15 minutes | 30 minutes | 1 hour | 1 ¼ hours | Not sure |
| --- | --- | --- | --- | --- |
| __ _1_ | __ _2_ | __ _3_ | __ _4_ | __ _5_ |

**Mental health-related health literacy**

**The following part is about whether you agree or disagree with certain statements about mental health. I will read the statements to you and you can tell me whether you totally agree, rather agree, neither agree nor disagree, rather disagree or totally disagree.**

*INT: Present show card 8*

**Q12**

- Please tick or cross one box in each row!

|  |  | Fully agree | Rather agree | Neither agree nor  disagree | Rather disagree | Fully disagree | Don’t know/ Refused |
| --- | --- | --- | --- | --- | --- | --- | --- |
| **Q12a** | Most people with mental health problems want to have paid employment. | __ _1_ | __ _2_ | __ _3_ | __ _4_ | __ _5_ | __ _8_ |
| **Q12b** | If a friend had a mental health problem, I know what advice to give them to get professional help. | __ _1_ | __ _2_ | __ _3_ | __ _4_ | __ _5_ | __ _8_ |
| **Q12c** | Medication can be an effective treatment for people with mental health problems. | __ _1_ | __ _2_ | __ _3_ | __ _4_ | __ _5_ | __ _8_ |
| **Q12d** | Psychotherapy (for example, talking therapy or counselling) can be an effective treatment for people with mental health problems. | __ _1_ | __ _2_ | __ _3_ | __ _4_ | __ _5_ | __ _8_ |
| **Q12e** | People with severe mental health problems can fully recover. | __ _1_ | __ _2_ | __ _3_ | __ _4_ | __ _5_ | __ _8_ |
| **Q12f** | Most people with mental health problems go to a health care professional to get help. | __ _1_ | __ _2_ | __ _3_ | __ _4_ | __ _5_ | __ _8_ |

**I will now read out some conditions and you tell me whether you think each of them is a type of mental illness.**

*INT: Present show card 8*

- Please tick or cross one box in each row!

|  |  | Fully agree | Rather agree | Neither agree nor  disagree | Rather disagree | Fully disagree | Don’t know/ Refused |
| --- | --- | --- | --- | --- | --- | --- | --- |
| **Q13a** | Depression | __ _1_ | __ _2_ | __ _3_ | __ _4_ | __ _5_ | __ _8_ |
| **Q13b** | Stress | __ _1_ | __ _2_ | __ _3_ | __ _4_ | __ _5_ | __ _8_ |
| **Q13c** | Schizophrenia | __ _1_ | __ _2_ | __ _3_ | __ _4_ | __ _5_ | __ _8_ |
| **Q13d** | Bipolar disorder (manic Depression) | __ _1_ | __ _2_ | __ _3_ | __ _4_ | __ _5_ | __ _8_ |
| **Q13e** | Drug addiction | __ _1_ | __ _2_ | __ _3_ | __ _4_ | __ _5_ | __ _8_ |
| **Q13f** | Grief | __ _1_ | __ _2_ | __ _3_ | __ _4_ | __ _5_ | __ _8_ |

| **General living conditions** |
| --- |

**We are now coming to the last part of the interview and I have a number of questions about your general living conditions.**

**Country of birth**

**Q14 In which country where you born?**

*INT: If other country, please write down name of country. If the country does not exist anymore, ask: ‘What is the country called nowadays?’*

*If Germany, please continue with* ***Q 16***

*If other, please continue with* ***Q15***

__ _1_ Germany

__ _2_ Other 🡪 [Q14a] Which other country? __________________

__ _8_ [Don´t know]

__ _9_ [Not specified]

**Language proficiency**

**Q15 How would you rate your German language skills?**

__ _5_ Very good

__ _4_ Good

__ _3_ Fair

__ _2_ Poor

__ _1_ Very poor

__ _8_ [Don’t know]

__ _9_ [Not specified]

**Education**

**Q16 What is your highest school qualification?**

| __ _1_ | No school qualification (yet) |
| --- | --- |
| __ _2_ | Lower secondary school qualification (after 9 or 10 years of schooling) |
| __ _3_ | A-Level or equivalent (after 12 or 13 years of schooling) |
| __ _4_ | Other school qualification 🡪 [Q16a] Which one?  __________________________________________________________________ |
| __ _8_ | [Don’t know] |
| __ _9_ | [Not specified] |

**Q17 What is your highest training/ further education qualification?**

| __ _1_ | Apprenticeship (professional company training) |
| --- | --- |
| __ _2_ | Vocational school, Trade school (vocational school training) |
| __ _3_ | Technical school (e.g., master technician school, professional or technical academy) |
| __ _4_ | University/Applied school of sciences degree (Bachelor, Masters) |
| __ _5_ | Other 🡪 [Q17a] Which one?  __________________________________________________________________ |
| __ _8_ | [Don’t know] |
| __ _9_ | [Not specified] |

**You have now answered all questions. Thank you very much!**

[Int6] Time (end of interview): __|__:__|__

[Int7] Interviewer comments:

|  |
| --- |
|  |
|  |
|  |
|  |
|  |
|  |
|  |
|  |
|  |
|  |
|  |

# Organisational Readiness Assessment


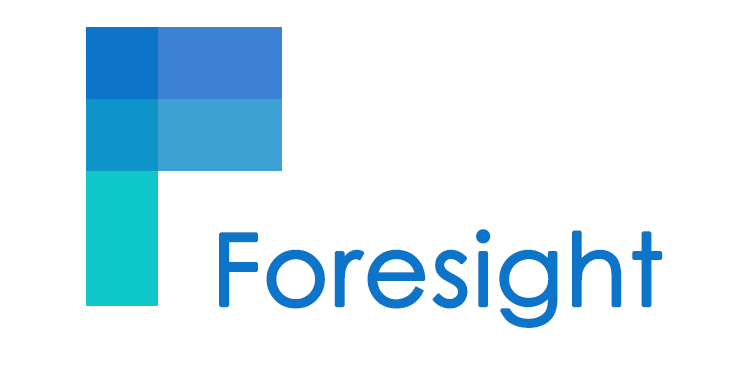


**Organizational Readiness Assessment**

**T1**

**Evaluation of intervention measures**

**Q1 What health promotion measures have taken place in your company in recent months as part of the FORESIGHT project?**

__ _1_Health workshop - Nutrition/Coronary heart disease module

__ _2_Health workshop - Mental health/depression module

__ _3_Health workshop - Physical exercise module

__ _4_Cooking activities

__ _5_Stress management/relaxation units

__ _6_Physical activity units

__ _7_None

__ _8_Other_________________

__ _(9) [_Don't know]/No answer

**Q2 How did you like the measures?**

|  |  | Very good | Good | Medium-moderate | Bad | Very bad | Does not apply |
| --- | --- | --- | --- | --- | --- | --- | --- |
| **F2aa-ac** | Health workshop - Nutrition module   - Content - The participants - Number of participants per date | __ _1_  __ _1_ | __ _2_  __ _2_ | __ _3_  __ _3_ | __ _4_  __ _4_ | ___5_  ___5_ | __ _8_  __ _8_ |
| **F2ba-bc** | Health workshop - Mental health module   - Content - The participants - Number of participants per date | __ _1_  __ _1_ | __ _2_  __ _2_ | __ _3_  __ _3_ | __ _4_  __ _4_ | ___5_  ___5_ | __ _8_  __ _8_ |
| **F2ca-cc** | Health workshop - Exercise module   - Content - The participants - Number of participants per date | __ _1_  __ _1_ | __ _2_  __ _2_ | __ _3_  __ _3_ | __ _4_  __ _4_ | ___5_  ___5_ | __ _8_  __ _8_ |
| **F2da-dc** | Cooking activities   - Content - The participants - Number of participants per date | __ _1_  __ _1_ | __ _2_  __ _2_ | __ _3_  __ _3_ | __ _4_  __ _4_ | ___5_  ___5_ | __ _8_  __ _8_ |
| **F2ea-ec** | Stress management/ relaxation sessions   - Content - The participants - Number of participants per date | __ _1_  __ _1_ | __ _2_  __ _2_ | __ _3_  __ _3_ | __ _4_  __ _4_ | ___5_  ___5_ | __ _8_  __ _8_ |
| **F2fa-fc** | Physical Activity units   - Content - The participants - Number of participants per date | __ _1_  __ _1_ | __ _2_  __ _2_ | __ _3_  __ _3_ | __ _4_  __ _4_ | ___5_  ___5_ | __ _8_  __ _8_ |
| **F2ga-gc** | Other   - Content - The participants - Number of participants per date | __ _1_  __ _1_ | __ _2_  __ _2_ | __ _3_  __ _3_ | __ _4_  __ _4_ | ___5_  ___5_ | __ _8_  __ _8_ |

**The following is about health literacy in your company. Health literacy is the motivation, knowledge and skills to find, understand, evaluate and apply health information in order to make health-related decisions in daily life. Health literacy is therefore a very broad concept. In short, this survey is about what is being done in your company to support the participants in the measures to behave in a way that promotes health.**

**If the survey refers to participants, this means all persons in your company who are financed by a Jobcenter measure.**

| **Knowledge about services to promote health literacy** |
| --- |

**In this section, we will first look at the services available to promote health literacy in your company . The term "offers" refers to all courses, programs, events or consultations that are intended to promote the health literacy of the participants in the measures.**

**Q1. Does your company currently offer programs to promote health literacy?**

Yes No

*Note: If "No", continue with question 5*

**Q2. Please name the three most important offers for promoting health literacy in your company.**

1. offer: _____________________________________________

2. offer: _____________________________________________

3. offer: _____________________________________________

**Q3. What do you think:**

**a) How many participants in your company have heard of the offers?**None  Few Some Many Most

*Note: If "Nobody", continue with question 4*

**b) How many participants in your company can name the offers?**

None  Few Some Many Most

**c) How many participants in your company are aware of the objectives of the measures?**

None  Few Some Many Most

**d) How many participants in your company know who the offers are aimed at?**

None  Few Some Many Most

**e) How many participants in your company know how the offers work?**

None  Few Some Many Most

**f) How many participants in your company know whether the offers to promote health literacy are successful (e.g. whether enough people take part, whether the participants are satisfied with the offers, whether the offers actually have a positive effect on dealing with health issues)?**

None  Few Some Many Most

**Q4. How many participants in your company do you think have misconceptions about the current offers (e.g. regarding participation conditions or procedure)?**

None  Few Some Many Most

| **Support from the operational management** |
| --- |

**The following section deals with the question of how company management perceives the issue of health literacy and whether it assumes responsibility in this area.**

**Q5**. **On a scale of 1-10, how much of an issue is the promotion of health literacy for the management in general, with 1 being "not an issue at all" and 10 being "a very important issue"?**

______ (enter value from 1-10)

**Q6**. **Does the management of really prioritize this issue?**

*Note: Give priority, e.g. by supporting offers to promote health literacy*

Yes Rather yes Rather no No

*Note: If no offers to promote health literacy among the long-term unemployed were mentioned (=question Q1 answered with "No"), continue with question 9*

**Q7. How does the management of show its support for the current measures?**

**a) Does the company management at least passively support the current offers (e.g. it thinks such offers are good, but does not participate in the planning)?**

No Rather no Partly, partly Rather yes Yes

**b) Is the company management involved in the planning or implementation of the measures?**

No Rather no Partly, partly Rather yes Yes

**c) Does the management support the allocation of resources for the current measures?**

*Note: Resources can be time, money, personnel, premises, etc.*

No Rather no Partly, partly Rather yes Yes

**d) Does the company management play an active role in the current offers (e.g. by emphasizing the importance of the offers in the company to the participants in the measures or by representing the topic externally)?**

No Rather no Partly, partly Rather yes Yes

**e) Is the management working towards maintaining the current services, e.g. by securing long-term financing?**

No Rather no Partly, partly Rather yes Yes

**f) Does the management regularly obtain an overview of the success of the current measures?**

No Rather no Partly, partly Rather yes Yes

**Q8**. **Would the company management support additional offers to promote health literacy?**

Yes Rather yes Rather no No

| **Implementation climate among the participants in the measures** |
| --- |

**The following section focuses on the attitudes of participants in the measures towards health literacy.**

**Q9**. **On a scale of 1-10, how much of an issue are health and health literacy in general among the participants in the measures, with 1 being "not an issue at all" and 10 being "a very important issue"?**

______ (enter value from 1-10)

**Q10**. **Do the participants in the measures actually prioritize this topic?**

*Note: Give priority, e.g. by participating in measures*

Yes Rather yes Rather no No

*Note: If no offers to promote health literacy were mentioned (=question Q1 answered with "No"), continue with question 13*

**Q11. How do participants show their support for the current measures?**

**a) How many participants at least passively support the current offers (e.g. they like such offers but do not take part in them)?**

Nobody Few Some Many Most

**b) How many participants are involved in the planning or implementation of the current measures?**

Nobody Few Some Many Most

**c) How many participants support the maintenance of the current measures?**

Nobody Few Some Many Most

**Q12**. **Would participants support additional offers?**

Yes Rather yes Rather no No

| **Knowledge about promoting health literacy** |
| --- |

**In the following, I would like to ask you for an assessment of the health literacy of the participants in the measures. These assessments may be personal impressions or estimates.**

**Q13**. **On a scale of 1-10, how well are the participants in the measures able to look after their own health? Where 1 stands for "not at all" and 10 for "very well".**

______ (enter value from 1-10)

**Q14.**

1. **On a scale from very easy to very difficult, how easy do you think it is for participants to judge when they should seek a second opinion from another doctor?**

Very easy Fairly easy Fairly difficult Very difficult

1. **On a scale from very easy to very difficult, how easy do you think it is for participants to make decisions about their illness with the information provided by the doctor?**

Very easy Fairly easy Fairly difficult Very difficult

1. **On a scale from very easy to very difficult, how easy do you think it is for participants to find information about support options for mental health problems such as stress or depression?**

Very easy Fairly easy Fairly difficult Very difficult

1. **On a scale from very easy to very difficult, how easy do you think it is for participants to judge whether the information about health risks in the media is trustworthy? (Television, Internet or other media)**

Very easy Fairly easy Fairly difficult Very difficult

1. **On a scale from very easy to very difficult, how easy do you think it is for participants to find information about behaviors that are good for their mental well-being?**

Very easy Fairly easy Fairly difficult Very difficult

1. **On a scale from very easy to very difficult, how easy do you think it is for participants to understand information in the media about how to improve their health?**

Very easy Fairly easy Fairly difficult Very difficult

| **Resources for offers (time, money, staff, premises, etc.)** |
| --- |

**The following section will focus on the resources available to promote health literacy/health promotion in the workplace***.*

*Note: If no offers to promote health literacy were mentioned (=question Q1 answered with "No"), continue with question 32*

**Q30**. **How are the current offers to promote health literacy financed?**

*Note: Multiple answers possible*

Job center

Course fees

Health insurance companies

Donations
 Other: _________________________________

**Q31. Is it likely that this funding will continue in the future?**

Yes Rather yes Rather no No

**Q32**. **What resources are available to you that could be used to promote health literacy in your company?**

**a) Are there any volunteers who could be deployed for offers?**

Yes, many Yes, some Yes, few No, none

**b) Are there any donations that could be used for offers?**

Yes, many Yes, some Yes, few No, none

**c) Is there any public funding that could be used for offers?**

Yes, many Yes, some Yes, few No, none

**d) Are there any experts who could support offers?**

Yes, many Yes, some Yes, few No, none

**e) Are there premises in which the offers can be carried out?**

Yes, many Yes, some Yes, few No, none

**Q33. Would the company management and the participants in the measures support the use of these resources for offers to promote health literacy?**

Yes No

*Note: If no offers to promote health literacy/health promotion among the long-term unemployed were mentioned (=question 1 answered with "No"), continue to "Demographic data"*

**Q34. On a scale of 1-5, where 1 is "no effort" and 5 is "great effort", how much effort is your company making** to **increase resources for health literacy programs?**

**a) Search for volunteers for current or future measures.**

1 2 3 4 5

None .... Very great efforts

**b) Search for external financial support (e.g. from health insurance companies, job centers) for current or future measures.**

1 2 3 4 5

None .... Very great efforts

**c) Write applications to receive funding for offers.**

1 2 3 4 5

None .... Very great efforts

**d) Recruitment of course instructors for further courses.**

1 2 3 4 5

None .... Very great efforts

**Q35**. **Have you heard of any applications or plans to increase resources for health literacy services ?**

Yes No

| **Demographic data & information on the company** |
| --- |

**Q36. Which gender do you belong to?**

Woman Man Divers

**Q37. How old are you?**

18-34 years

35-49 years

50-64 years

65+ years

**Q38. Company size**

<10 participants

10-30 participants

>30 participants

**Q39. Your area of activity in the company?**

Social worker

Management

Participants
 Other: _________________________________
